# Antimicrobial Properties of Palladium and Platinum Nanoparticles: A New Tool for Combating Food-Borne Pathogens

**DOI:** 10.3390/ijms22157892

**Published:** 2021-07-23

**Authors:** Ondrej Chlumsky, Sabina Purkrtova, Hana Michova, Hana Sykorova, Petr Slepicka, Dominik Fajstavr, Pavel Ulbrich, Jitka Viktorova, Katerina Demnerova

**Affiliations:** 1 Department of Biochemistry and Microbiology, University of Chemistry and Technology, Technicka 5, 166 28 Prague 6, Czech Republic; Sabina.Purkrtova@vscht.cz (S.P.); Hana.Michova@vscht.cz (H.M.); Hana.Sykorova@vscht.cz (H.S.); pavel.ulbrich@vscht.cz (P.U.); jitka.prokesova@vscht.cz (J.V.); katerina.demnerova@vscht.cz (K.D.); 2 Department of Solid State Engineering, University of Chemistry and Technology, Technicka 5, 166 28 Prague 6, Czech Republic; Petr.Slepicka@vscht.cz (P.S.); dominik.fajstavr@vscht.cz (D.F.)

**Keywords:** palladium nanoparticles, platinum nanoparticles, antimicrobial properties, food-borne pathogens, minimum inhibitory concentrations, acute cytotoxicity

## Abstract

Although some metallic nanoparticles (NPs) are commonly used in the food processing plants as nanomaterials for food packaging, or as coatings on the food handling equipment, little is known about antimicrobial properties of palladium (PdNPs) and platinum (PtNPs) nanoparticles and their potential use in the food industry. In this study, common food-borne pathogens *Salmonella enterica* Infantis, *Escherichia coli*, *Listeria monocytogenes* and *Staphylococcus aureus* were tested. Both NPs reduced viable cells with the log_10_ CFU reduction of 0.3–2.4 (PdNPs) and 0.8–2.0 (PtNPs), average inhibitory rates of 55.2–99% for PdNPs and of 83.8–99% for PtNPs. However, both NPs seemed to be less effective for biofilm formation and its reduction. The most effective concentrations were evaluated to be 22.25–44.5 mg/L for PdNPs and 50.5–101 mg/L for PtNPs. Furthermore, the interactions of tested NPs with bacterial cell were visualized by transmission electron microscopy (TEM). TEM visualization confirmed that NPs entered bacteria and caused direct damage of the cell walls, which resulted in bacterial disruption. The in vitro cytotoxicity of individual NPs was determined in primary human renal tubular epithelial cells (HRTECs), human keratinocytes (HaCat), human dermal fibroblasts (HDFs), human epithelial kidney cells (HEK 293), and primary human coronary artery endothelial cells (HCAECs). Due to their antimicrobial properties on bacterial cells and no acute cytotoxicity, both types of NPs could potentially fight food-borne pathogens.

## 1. Introduction

Food-borne pathogens are among the most common causes of bacterial contamination in food processing plants [1,2,3]. They predominantly exist as communities of sessile cells that develop as biofilms [4]. Biofilm formation as a microbial growth strategy offers numerous advantages to microorganisms in comparison to planktonic lifestyle, such as better protection from hostile environmental hazards, higher resistance to antimicrobial agents, bacteriophages and other hostile environmental conditions [5,6]. Biofilm development is commonly considered to appear in four main stages: (I) bacterial attachment to a surface, (II) microcolony formation when bacteria initiate to produce excessive extracellular matrix, (III) biofilm maturation and (IV) detachment (also termed dispersal) of bacteria which may then colonize new areas [7]. To enhance food safety, the inhibition of initial bacterial attachment is an essential strategy to prevent biofilm formation on food processing surfaces [8,9]. In the next stages, bacteria generate the extracellular matrix consisting of extracellular polymeric substances (EPSs) such as exopolysaccharides, extracellular DNA (eDNA), proteins and lipids which contribute to cell survival and the resistance of the biofilm mass to environmental conditions. These EPSs directly influence a variety of biofilm physico-chemical characteristics, such as its porosity, density, water content, permeability, absorption, hydrophobic properties, mechanical resistance and other properties [10,11,12]. 

In spite of intensive efforts to improve sanitization strategies, microbial contamination containing antimicrobial-resistant food-borne pathogens persists as a problem in the food industry [8,13]. Therefore, novel strategies must be explored in the effort to inhibit bacterial colonization and reduce the risk of associated potential food-borne diseases, which is an increasingly common public health problem [1,14,15]. Novel strategies for antimicrobial agents could be found in the field of nanotechnology. An earlier report exhibited the advantage of the use of metallic NPs over other commonly employed antimicrobials, as they do not differentiate between resistant and susceptible bacteria [16]. In addition, they disturb the biofilm integrity by interacting with EPSs, eDNA, proteins, and lipids of biofilms [16,17]. The interactions of NPs with bacteria induce oxidative stress via reactive oxygen species which damage bacterial cell envelopes, cell membranes, cellular structures and biomolecules [16,17,18,19]. Thus, nanoparticles may be particularly advantageous in treating bacterial infection, preventing infections in a form of antibacterial coatings of implantable devices and medicinal materials, the promotion of wound healing, or as antibiotic delivery systems to treat diseases [17,20]. On the other hand, different types of NPs have distinct disadvantages, such as a short shelf life, poor stability and insufficiently explored cytotoxicity [17,21]. 

In the food industry, nanotechnology is already being used, for example, to generate antimicrobial nanomaterials commercially available as food packaging, or as antimicrobial coatings on the food handling equipment [22,23]. Materials used for antimicrobial application may consist of polymers, organic/inorganic nanoparticles, plastics or ceramics [18]. Various syntheses have been developed to obtain NPs with the desired quality while avoiding the aggregation, oxidation, and inactivation of the NPs during synthesis [24]. Unfortunately, chemical synthesis involves toxic chemicals in the synthesis protocol. To avoid the presence of chemical agents associated with environmental toxicity, eco-friendly synthesis approaches are in demand [25]. For instance, earlier study demonstrated a robust simple but rapid green synthesis of gold nanoparticle–alginate biohydrogel, using thermostable nisin while retaining the strong antimicrobial activity [24]. 

Besides the food industry, nanoparticles are nowadays broadly used in many other areas such as agriculture (nano-sized pesticides, herbicides, fungicides, fertilizers and sensors for crop cultivation and harvesting, pathogen detection and soil parameters [26]), medicine (nanomaterials functionalized with AgNO_3_ and CHX [27], including nanofibers-based sensors for clinical monitoring [28]), biotechnology (mesoporous SiO_2_ biosensors for enzyme immobilization [29]), cosmetics (nanotechnology-based sun creams, cosmetic powders, nanoemlusions and micelles [30]) and renewable energies (wind and geothermal power, energy storage, lighting and hydrogen fuel cells [31]).

Further, nanomaterials may be created from pure metals, or from their composites, with variable sizes and shapes [17,32,33]. The alteration of NPs’ size and shape changes their properties on the atomic level and has the potential to design their optimal physicochemical, optical and biological properties for various applications [32,34]. The distinctive physicochemical and optical properties of nanoparticles allow the design of systems with high sensitivity, large surface areas, special surface effects, high functional density, catalytic effects and enhanced optical emission [34,35]. In addition, variable NP sizes and shapes are likely to influence particle transport behavior in biological systems, as well as how cells sense and respond to the particle [36].

In our previous study, we reported the antimicrobial properties of gold (AuNPs) and silver nanoparticles (AgNPs) [37]. In this follow-up study, we aimed to examine the potential antimicrobial properties of palladium (PdNPs) and platinum (PtNPs) nanoparticles and their mechanism of action. While PtNPs are believed to induce the intracellular hyperproduction of ATP and oxygen radicals, in turn causing bacterial growth inhibition, DNA damage and bacteriotoxic effects [38,39,40], the precise mechanism of action of PdNPs has not been reported to date. Further, we investigated the acute cytotoxicity of NPs on selected cell lines to elucidate the potential impacts of NP exposure on the human population, as there is a gap in the current literature regarding their nanotoxicity [21,22,41]. 

**Figure 1:**
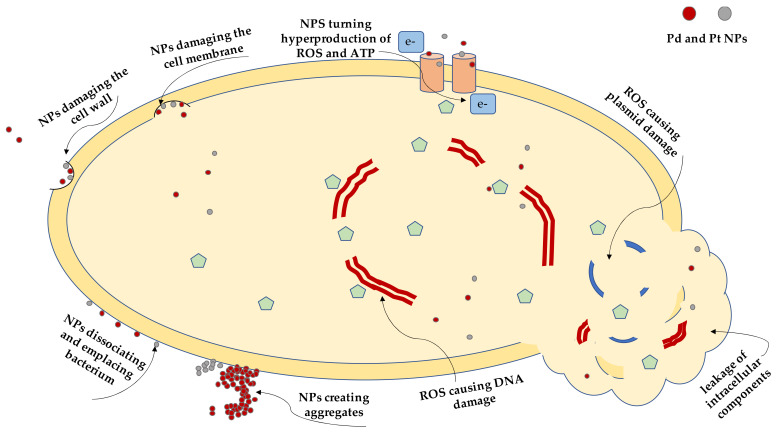
A schematic illustration of possible mechanism of action of palladium nanoparticles (PdNPs) and platinum nanoparticles (PtNPs) on a bacterial cell and its components.

In the presented study, four significant food-borne pathogens (*Salmonella enterica*, *Escherichia coli*, *Listeria monocytogenes* and *Staphylococcus aureus*) were selected to test the antimicrobial properties of PdNPs and PtNPs. These pathogens are well known for being potential biofilm-related sources of food-borne diseases with significant effects on human health and adverse economic impacts for the food industry. The effectiveness of the NPs was assessed by determining their minimum inhibitory concentrations needed for the inhibition of bacterial growth, biofilm formation, metabolic activity, and for biofilm reduction. TEM imaging was used to visualize the interactions of metallic NPs with planktonic cells and potentially reveal their mechanisms of action, which is schematically illustrated in Figure 1. The acute cytotoxicity of individual NPs was verified in vitro.

## 2. Results

Ten concentrations of NPs were tested to determine the minimum inhibitory concentration for planktonic growth, and six concentrations were applied for preformed biofilms (as the lowest concentration were known to be ineffective). The MIC was defined as the lowest substance concentration able to inhibit at least 80% of microbial growth (MICPC_80_ for planktonic cells, MICBC_80_ for further growth of biofilm cells), inhibit 80% of metabolic activity (MICBM_80_ for biofilm metabolic activity, MICMPB_80_ for metabolic activity of preformed biofilm), prevent biofilm formation by at least 80% (MICBF_80_ for biofilm formation), or reduce a preformed biofilm by at least 80% (MICBR_80_ for biofilm reduction). The results of MICs, log_10_ CFU reduction and inhibitions are summarized in Table 1, Table 2, Table 3, Table 4, Table 5 and Table 6. Complete data are provided in Supplementary Materials. 

### 2.1. The Effect of Palladium Nanoparticles 

According to the A_620_, planktonic growth was only inhibited in the case of two *E. coli* strains (683/17 and 693/17) where the MICPC_80_ was determined as 22.25 mg/L. For the other strains, MICPC_80_ values could not be determined, as they were higher than the maximal tested concentration (22.25 mg/L). The average A_620_ inhibition ranged from 28.6 to 92% (Table 1 and Table S1 and Figure S1). Similarly, the MTT values for biofilm metabolic activity (MICBM_80_) could not be determined neither for Gram-positive nor Gram-negative bacteria. The maximum inhibition of metabolic activity ranged from 3.3 to 52.1% (Table 1). For preformed biofilm, PdNPs were able to prevent further growth of biofilm cells and inhibit their metabolic activity in all strains (Table 3 and Table S2 and Figure S4). In addition, PdNPs were able to prevent the biofilm formation of both *S. aureus* strains and reduce biofilms of *S. aureus* 816 and both strains of *S*. Infantis (Table 1 and Table 3, Figure S2, Figure S3, Figure S5 and Figure S6). 

### 2.2. The Effect of Platinum Nanoparticles 

The results for PtNPs resemble those for PdNPs. In accordance with A_620,_ the MICPC_80_ values could not be determined, as they were higher than the maximal tested concentration (50.5 mg/L) for all strains. The average A_620_ inhibition ranged from 28.9 to 77.8% (Table 2, Table S3 and Figure S7). For biofilm metabolic activity (MICBM_80_), the MTT reduction assay evaluated maximum inhibition which ranged from 5.8 to 64.3% (Table 2). Thus, MICBM_80_ values could not be determined for any tested strains. However, preformed biofilm PtNPs were able to inhibit further growth of biofilm cells and inhibit their metabolic activity for all tested strains (Table 4 and Table S4 and Figure S10). Furthermore, PtNPs were able to prevent biofilm formation to the same degree as PdNPs for *S*. *aureus* 816 and were able to reduce preformed biofilm in both *S*. *aureus* strains (Table 2 and Table 4, Figure S8, Figure S9, Figure S11 and Figure S12).

### 2.3. Colony Plate Counting and Inhibitory Rate Method

PdNPs’ and PtNPs’ effects on bacterial growth were further studied by the colony plate counting and calculation of inhibitory rate (Table 5 and Table 6). The log_10_ CFU reduction ranged from of 0.3–2.4 (PdNPs) and 0.8–2.0 (PtNPs), which represent the complete inhibition of bacterial growth at the maximal tested concentration (22.25 mg/L PdNPs or 50.5 mg/L PtNPs), except for *L. monocytogenes* 149 when PdNPs were applied (Table 5). The average inhibitory rates ranged from 55.2 to 99% in the case of PdNPs (Table 5) and from 83.8 to 99% in the case of PtNPs (Table 6).

### 2.4. Transmission Electron Microscopy Imaging

**Figure 2:**
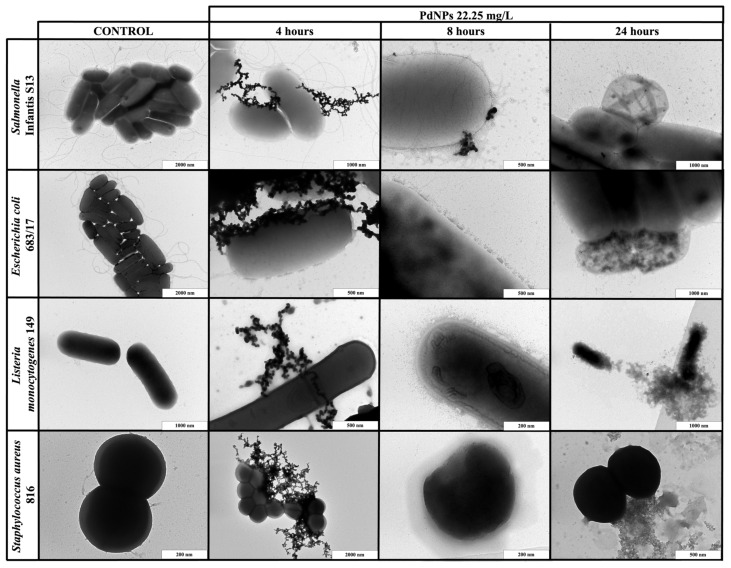
TEM visualization of the interactions between palladium nanoparticles (PdNPs) and planktonic cells of *S.* Infantis S13, *E. coli* 683/17, *L. monocytogenes* 149 and *S. aureus* 816 after 4, 8 and 24 h exposure.

**Figure 3:**
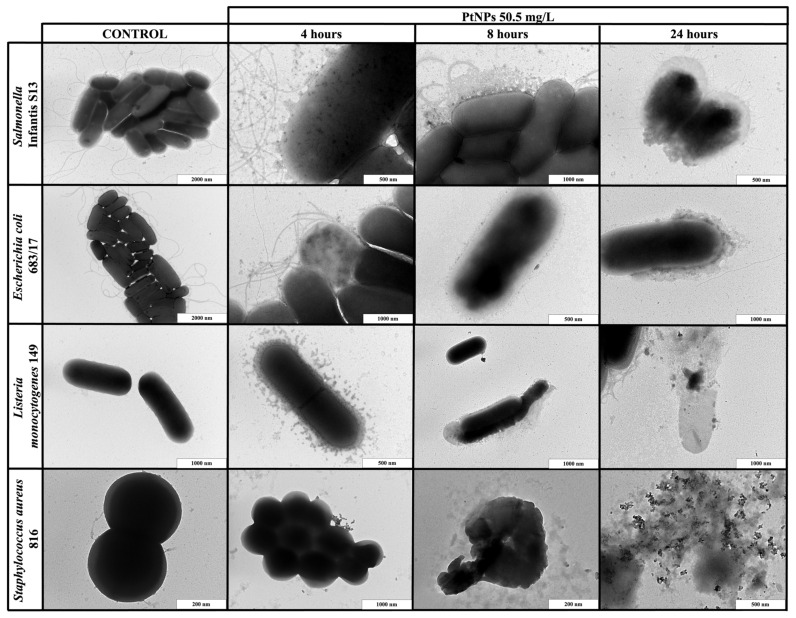
TEM visualization of the interactions between platinum nanoparticles (PtNPs) and planktonic cells of *S.* Infantis S13, *E. coli* 683/17, *L. monocytogenes* 149 and *S. aureus* 816 after 4, 8 and 24 h exposure.

To better understand the mechanism of action, selected bacterial strains were exposed to the highest effective concentration of the metallic NPs for different durations (for 4, 8 and 24 h), and were then observed with TEM. The application of NPs resulted in bacterial disruption and leakage of intracellular components (Figure 2 and Figure 3). These observations were not detected in the planktonic cells without NPs.

When PdNPs (22.25 mg/L) or PtNPs (50.5 mg/L) were applied to the planktonic cells, the results were exposure-dependent. The shortest exposure (4 h) did not result in any bacterial changes, but PdNPs created huge aggregates around both Gram-positive and Gram-negative bacterial cells, while PtNPs only formed small aggregates. After 8 h of exposure, both types of NPs dissociated and entered bacteria or were emplaced or partially aggregated around the cells, causing direct damage of the cell walls. The longest exposure (24 h) resulted in complete bacterial disruption and leakage of intracellular components for both types of NPs.

### 2.5. Acute Cytotoxicity of Metallic Nanoparticles

The cytotoxic effect of metallic NPs on HRTECs, HaCat, HDFs, HEK 293, HCAECs was evaluated by a resazurin assay over 72 h to determine the concentration that halved the cellular viability (IC_50_). The IC_50_ (mg/L) values are demonstrated in Table 7. No IC_50_ values were obtained for both PdNPs and PtNPs, because they did not cause any acute cytotoxicity in a concentration range up to 4.45 mg/L (PdNPs) and 10.1 mg/L (PtNPs).

## 3. Discussion

In this work, two types of metallic NPs (PdNPs and PtNPs) were tested for their ability to inhibit cell growth, prevent biofilm formation, and to reduce the biofilm mass of four selected bacterial food-borne pathogens (Gram-positive *L*. *monocytogenes*, *S*. *aureus* and Gram-negative *E*. *coli*, *S.* Infantis). The highest concentrations applied in this study (PdNPs 44.5 mg/L and PtNPs 101 mg/L) were prepared by using the cathodic sputtering approach, which requires a specific time deposition.

PdNPs and PtNPs were characterized by TEM and high-resolution TEM (round shape, size 4–6 nm). Both NPs exhibited greater antimicrobial effects on further growth of biofilm cells and the metabolic activity of preformed biofilm than on planktonic cells. Nevertheless, further investigations, such as colony plate counting and TEM visualization confirmed their antimicrobial properties. These effects were mainly observed at the highest concentrations applied (PdNPs 22.25–44.5 mg/L, PtNPs 50.5–101 mg/L), which may cause significantly higher expense for an application in food processing plants. In a previous study [37], we demonstrated a similar result for gold and silver NPs.

According to our review of the literature, the antimicrobial activity of PdNPs and PtNPs against *L*. *monocytogenes* and *Salmonella* Infantis has not been reported to date. A small handful of studies described the antimicrobial activity of PdNPs and PtNPs for other bacterial species [38,42,43,44]. A study of Adams et al. [42] demonstrated greater antimicrobial activity of PdNPs (size 2 nm) at concentrations as low as 2.5 nM against Gram-positive *S. aureus* compared to Gram-negative *E. coli*. Nevertheless, the antimicrobial effect for Gram-negative *E. coli* required higher concentrations of PdNPs and longer exposure times before an inhibitory growth effect became evident, which corresponded with our current work. Their study further confirmed that the antimicrobial activity of NPs is size-dependent, as the most effective NPs size was established as <1 nm. However, NPs < 1 nm may possess relatively high ecological risk if they enter the environment. Therefore, comparatively “large” NPs were studied firstly. To the best of our knowledge, NP size could be successfully altered by adjusting the concentration of PEG or adding certain additives. This size-dependent correlation with antimicrobial activity was also demonstrated in studies describing that NP size plays a major role in their antimicrobial activity against both Gram-positive and Gram-negative bacteria [43,44]. For instance, NPs bigger than 5 nm only interact with the cell membrane, while smaller NPs have the potential to enter bacteria. As well as for entering bacteria, TEM visualization further confirmed interactions enable better binding of NPs to the bacterial cell wall. This observation was detected in our earlier study [37] and is explained by Slavin et al. [45], who described this affinity for a wide spectrum of bacteria.

Similarly, the potential antimicrobial activity of PtNPs has only been demonstrated in a few studies. Hashimoto et al. [38] reported the antimicrobial effect of PtNPs at concentrations of 400 mg/L with an NP size < 5 nm. According to their work, PtNPs exhibited an inhibitory effect on biofilm formation. Our study only indicated an inhibitory effect on biofilm formation for *S. aureus* 816.

As previously mentioned, the discrepancies of published results may be explained by differences in the nanoparticle sizes tested, nanoparticle concentrations or shapes, or by different testing conditions [37]. Additionally, there is limited understanding of the potential nanotoxicity associated with the use of metallic NPs. To date, many studies have explored the potential impacts of NP exposure on the human population, associated safety concerns, and environmental concerns [21,22,41]. There are only a few studies that offer useful conclusions regarding the safety of NPs [41]. Furthermore, it was demonstrated that it is not possible to make a single overarching recommendation concerning the safety of all nanoparticle types [21]. Instead, the toxicity of NPs should be judged on a case-by-case basis. Our results report no acute cytotoxic activity of PdNPs and PtNPs. However, each type of NP should be thoroughly investigated, especially regarding their composition, size and dose, before guaranteeing their safe application in the food industry [22].

For future studies, there needs to be a renewed focus on evaluating antimicrobial activity as a function of NP size. Although NPs seem to be a theoretically promising tool for bacterial growth combat in food processing plants, it may be difficult to strike a balance between their efficient use and toxicity. Therefore, it is very important to continue testing the efficacy and safety of NPs, in all their permutations, in the greater effort to find the most convenient and safe surface strategy required in the food industry.

## 4. Materials and Methods

### 4.1. Chemicals and Reagents

The liquid media used for the cultivation of bacteria were Brain Heart Infusion (BHI) or Tryptone Soya Broth supplemented with 1% glucose (TSB + 1% Glc). The following solid media were used: selective–diagnostic agars Baird–Parker (BP) agar, agar Listeria according to Ottaviani and Agosti (ALOA), Xylose Lysine Deoxycholate (XLD) agar and Tryptone Bile X-glucuronide (TBX) agar and non-selective plate count agar (PCA). All media were purchased from Merck (Darmstadt, Germany) or Oxoid (Hampshire, United Kingdom). Dimethyl sulfoxide (DMSO), 96% ethanol and glucose were purchased from Penta (Prague, Czech Republic). The chemicals 3-(4,5-dimethyl-2-thiazolyl)-2,5-diphenyl-2H tetrazolium bromide (MTT), crystal violet, polyethylene glycol (PEG) 600 and sodium dodecyl sulfate (SDS) were purchased from Sigma Aldrich (Burlington, MA, USA). Palladium and platinum of 99.9999% purity used for NP preparation were both purchased from Safina (Vestec, Czech Republic). Phosphate buffer solution (PBS) was bought from Lonza (Kourim, Czech Republic). Washing solution was prepared by mixing 40% DMSO solution, 1× PBS and dissolving SDS to a final concentration of 160 mg/mL. Dulbecco’s modified Eagle’s medium (DMEM), trypsin/EDTA solution, antibiotic mixture (penicillin and streptomycin), fetal bovine serum (FBS), resazurin sodium salt were purchased from Merck (Kenilworth, NJ, USA). ProxUp Basalmedium with ProxUp supplements were purchased from Evercyte (Vienna, Austria). Vascular cell basal medium and an endothelial cell growth kit were purchased from ATCC (Manassas, VI, USA).

### 4.2. Preparation of Metallic Nanoparticles

**Figure 4:**
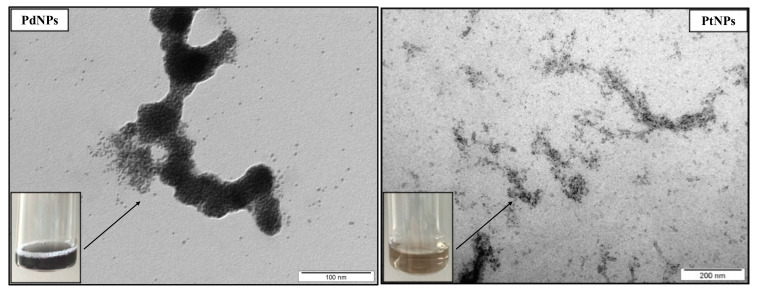
TEM images of palladium nanoparticles (PdNPs) and platinum nanoparticles (PtNPs).

**Figure 5:**
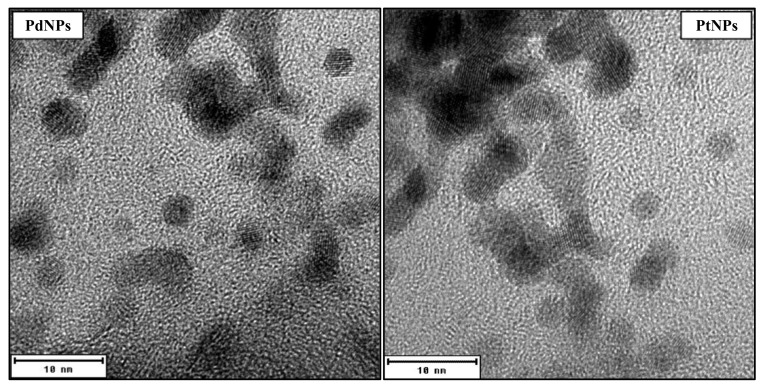
HR-TEM images of round shape palladium nanoparticles (PdNPs) and platinum nanoparticles (PtNPs) with size 4–6 nm.

Metallic NPs (PdNPs and PtNPs) were prepared by the Department of Solid State Engineering, University of Chemistry and Technology in Prague, by cathodic sputtering using a BAL-TEC SCD 050 nebulizer, loaded directly into 2 mL of polyethylene glycol pipetted in a Petri dish. The deposition was carried out under constant conditions: room temperature, argon pressure in 8Pa chamber, current 30 mA, electrode gap 50 mm and time deposition 1000 s. After spraying, the nanoparticulate polyethylene glycol was immediately diluted with 18 mL of distilled water, i.e., 1:9 by volume (PEG:H_2_O). NPs were characterized by TEM (Figure 4) and HR-TEM (Figure 5) as being of a round shape with size of 4–6 nm.

### 4.3. Bacterial Strains

For Gram-positive species, two strains of *Staphylococcus aureus* (*S. aureus* 816 and 1241) and two strains of *Listeria monocytogenes* (*L. monocytogenes* 149 and 164) were tested. For Gram-negative species, four strains of *Escherichia coli* (*E. coli* 683/17, 693/17, 815 and 859) and two strains of *Salmonella enterica* Infantis (S13 and S59) were tested. All strains, except for *E. coli* 683/17 and 693/17, were isolated at the Department of Biochemistry and Microbiology at the University of Chemistry and Technology in Prague, Czech Republic and the National Institute of Public Health, Brno, Czech Republic. The *E. coli* 683/17 and 693/17 were provided by the Veterinary Research Institute Brno, Czech Republic. All tested strains originated from food processing plants (Table 8).

### 4.4. Cell Lines and Cell Cultures

To test the acute cytotoxicity of the nanoparticles, the following cell lines were used: primary human renal tubular epithelial cells (HRTECs-CHT-003-0002; Evercyte, Vienna, Austria); human keratinocytes (HaCat-C0055C; Thermo Fisher Scientific, Waltham, MA, USA); human dermal fibroblasts (HDFs-106-05A; Merck, Kenilworth, NJ, USA); human epithelial kidney cells (HEK 293-CRL-3249; ATCC, Manassas, VI, USA); primary human coronary artery endothelial cells (HCAECs-PCS-100-020; ATCC, Manassas, VI, USA).

### 4.5. Bacterial Stock Cultures Preparation

Isolates were refreshed from a deep-frozen aliquot by inoculating one loopful on the following agar plates—ALOA for *L. monocytogenes*, BPA for *S. aureus*, XLD for *Salmonella* Infantis and TBX for *E. coli*. Strains were incubated at 37 °C for 24 h. Grown cultures were stored at 4 °C for up to one month and used for inoculum preparation.

### 4.6. Inoculum Preparation and Preparation of Dilution Series for Metallic Nanoparticles

A single colony from an agar plate was inoculated into 2 mL of BHI and incubated at 37 °C overnight. To obtain the starting cultures, strains of *S. aureus*, *L. monocytogenes* and *E. coli* were centrifuged (6000 *g*, 10 min) and the resulting pellet was resuspended in 2 mL of TSB + 1% Glc, which was previously shown as an optimal medium for their biofilm growth [37]. For *Salmonella* strains, the overnight grown culture was used directly as the starting culture, since the same medium (BHI) was used for inoculum preparation [37]. In all cases, inoculum was prepared by mixing the chosen fresh medium for biofilm formation with the starting culture to reach a bacterial density of 0.5 McFarland standard. A dilution series of the tested antimicrobial substances (metallic NPs) were prepared by diluting the substances in appropriate culture medium (BHI, TSB + 1% Gl) in a 1:1 ratio. The concentration range for PdNPs was 0.05–44.5 mg/L and for PtNPs, 0.1–101 mg/L. The highest available concentration of PdNPs and PtNPs was used only for biofilm reduction testing, where NPs were directly applied to a preformed biofilm. Ten different concentrations of NPs were prepared as two-fold dilution series by mixing the appropriate concentrations in the ratio 1:1.

### 4.7. Determination of Minimum Inhibitory Concentrations

The minimum inhibitory concentrations were determined as described by Chlumsky et al. [37]. Briefly, 75 µL of inoculum (0.5 McFarland) were transferred into a pre-sterilized polystyrene 96-well flat-bottomed microtiter plate in three replicates and were then carefully mixed with 75 µL of a test substance at a particular concentration. For a positive control of bacterial growth, the inoculum was mixed with pure sterile medium. Furthermore, sterile medium was included in the plate as a marker of potential microbial contamination.

All inhibitions and inhibitory rate were calculated using the modified formula of Qin et al. [46] below (Equations (1) and (3)):

(1) $$A_{620\left(595\right)}\;\mathrm{inhibition}\;\left(\%\right)\;=\frac{A_{620\left(595\right)}\;\left(\mathrm{control}\right)\;-{\;A}_{620\left(595\right)}\;\left(\mathrm{nanoparticles}\right)}{A_{620\left(595\right)}\;\left(\mathrm{control}\right)}\times 100$$

where A_620(595)_ (control) is the absorbance of bacterial suspension/biofilm itself and A_620(595)_ (nanoparticles) is the absorbance of the bacterial suspension/biofilm with the added PdNPs or PtsNPs.

The MICs represent the minimum concentrations which resulted in at least 80% inhibition of growth (MICPC_80_, MICBC_80_), metabolism (MICBM_80,_ MICMPB_80_) and biofilm formation (MICBF_80_), or resulted in to at least 80% reduction in preformed biofilms (MICBR_80_). When the minimum inhibitory concentration could not be determined, the MIC was established as >44.5 mg/L for PdNPs or >101 mg/L for PtNPs.

#### 4.7.1. Evaluation of Planktonic Cells Growth

For the determination of MICPC_80_, the optical density of the content of the microtiter plates was measured spectrophotometrically at 620 nm before and after 24 h of cultivation at 37 °C (25 °C for *S.* Infantis strains [37]). The difference of A_620_ was considered as a measure of the ability of planktonic cells to grow in the presence of the tested NPs and was used to determine MICPC_80_. After cultivation, the biofilm was quantified using the crystal violet assay (4.7.2.) or tested for metabolic activity (4.7.3.).

#### 4.7.2. Quantification of Biofilm Formation

For the determination of MICBF_80,_ biofilms were quantified using crystal violet staining [37]. The wells of microtiter plates with grown bacterial culture were washed five times with 200 µL of distilled water using an automated microtiter plate washer and dried at room temperature for 45 min. Then, 150 µL of 0.1% crystal violet solution in sterile distilled water was added to each well, staining the biofilm for 45 min. After staining, the wells were washed again as mentioned above. Then, 200 µL of 96% ethanol was added for 15 min to elute the stain from the biofilm. Next, 100 µL of eluted solutions was transferred into a new microtiter plate and measured spectrophotometrically at 595 nm.

#### 4.7.3. Evaluation of Metabolic Activity

The determination of MICBM_80_ was estimated by using the MTT (thiazyl tetrazolium bromide) reduction assay. The bacterial cultures in a microtiter plate were drained off and the wells were washed twice with 200 µL of PBS. Next, 80 µL of glucose solution (57.4 mg/mL) and 70 µL of MTT solution (1 mg/mL) were added into each well and mixed. The microtiter plate was wrapped in tinfoil and incubated for 2 h at 37 °C (25 °C for *S.* Infantis). Then, 100 µL of washing solution was added and the microtiter plate was statically incubated for at least 30 min at 37 °C (25 °C for *S*. Infantis) in order to dissolve the preformed formazan. Next, the solution was mixed by pipetting five times and 100 µL of each solution was transferred into a new microtiter plate and spectrophotometrically assessed at 595 nm.

#### 4.7.4. Evaluation of Nanoparticles Effect on Preformed Biofilms

For the determination of MICBR_80_, 100 µL of inoculum (0.5 McFarland) was added into a microtiter plate well in three replicates for each strain and concentration. The plate was incubated for 18 h at 25 °C (*S.* Infantis) or at 37 °C (other species) to allow the cells to form biofilms. The plate was then washed four times with 200 µL of sterile distilled water by manual pipetting in order to avoid cross-contamination occurring when using the plate washer. Then, 100 µL of the tested substances diluted with medium was added onto the preformed biofilms. Positive and sterility controls were included in the experiment. The resulting suspensions were measured spectrophotometrically at 620 nm before and after following 24 h of cultivation at 37 °C (25 °C for *S.* Infantis). The difference of A_620_ was considered as a measure of the ability of biofilm cells to grow in the presence of tested NPs and was used for the determination of MICBC_80_. After the cultivation, the biofilm was quantified using the crystal violet assay (MICBR_80_) or tested for biofilm metabolic activity (MICMPB_80_) as described above.

### 4.8. Evaluation of Growth Inhibition Using the Plate Counting Agar

The highest concentrations of metallic NPs (44.5 mg/L PdNPs or 101 mg/L PtNPs) were mixed with individual bacterial suspension (10^7^ to 10^8^ CFU/mL) in the ratio 1:1 and cultivated for 24 h at 37 °C with shaking of 135 rpm. Before and after cultivation, the suspensions were serially decimally diluted and compared by quantifying their CFU/mL. The three most diluted suspensions were applied in 20 μL droplets on a plate count agar (PCA, Oxoid, Cheshire, UK) in two parallels and incubated for 24 h at 37 °C. After the cultivation, the grown bacterial colonies were counted and quantified according to Lencova et al. [27]. Four independent replicates were performed for each bacterial strain with specific metallic nanoparticles. Bacterial suspensions without any added NPs were used as controls.

From the CFU/mL determination, log_10_ CFU reduction was assessed according to Equation (2) (log_10_ CFU reduction expresses the difference between bacterial growth in the control and the suspension with the PdNPs or PtNPs) [47]. The inhibitory effect was calculated using the modified formula below (Equation (3)).

*log_10_ CFU reduction* = log_10_ control − log_10_ nanoparticles (2)

where log_10_ control is the number of bacterial cells in the suspension itself and log_10_ nanoparticles is the number of bacterial cells in the suspension with the added PdNPs or PtsNPs.

(3) $$Inhibitory\;rate\;\left(\%\right)\;=100\times \frac{\log_{10}\;\mathrm{control}\;-{\;\log}_{10}\;\mathrm{nanoparticles}}{\log_{10}\;\mathrm{control}}$$

where CFU (control) is the number of CFU/mL in the bacterial suspension itself and CFU (nanoparticles) is the number of CFU/mL in the bacterial suspension with the added PdNPs or PtsNPs.

### 4.9. Transmission Electron Microscopy Imaging

The interactions between tested metallic NPs and planktonic cells were visualized by TEM. The volume of 0.75 mL of inoculum (10^7^ or 10^8^ CFU/mL) was added into a 2 mL centrifuge tube and mixed with 0.75 mL metallic NPs of selected concentration or 0.75 mL of sterile medium (control). After cultivation (37 °C for 4, 8 and 24 h) in a shaking incubator, a drop of a bacterial culture suspension was deposited on a copper carbon-coated electron microscopic grid and incubated at room temperature for about 10 min. After that, the excess of liquid was removed by filter paper and the grid was quickly rinsed with distilled water. The grid was then deposited into a solution of 1% sodium silicotungstate (pH 7.4) and negatively stained for about 10 sec. After the staining, the grid was left to dry and subsequently inserted into the TEM column JEOL JEM-1010 (JEOL Ltd., Tokyo, Japan) operated at 80 kV at various magnifications. The micrographs were recorded by SIS Megaview III CCD camera and analyzed using AnalySIS v3.2 software (Olympus Soft Imaging Systems, Münster, Germany).

### 4.10. Cytotoxicity Assay

The cell lines were maintained in a proper medium—HaCat, HDFs and Hek 293 in DMEM; HRTECs in VCB; HCAECs in ProxUp. The cytotoxicity experiment was realized according to Tran et al. [48]. Briefly, the cells were counted by a Cellometer Auto T4 (Nexcelom Bioscience, Lawrence, MA, USA) and the cell suspension containing a cell density of 10^5^ cells/mL was split into the 96-well plate, 100 µL per well. The plates were then incubated for 24 h at 37 °C in humidified atmosphere of 5% CO_2_. Then, the plates were washed three times with PBS and the tested NPs diluted in the respective medium were added using a binary serial dilution. After 72 h of incubation, the cell viability was tested by a resazurin assay. The fluorescence was measured by a SpectraMax i3x microplate reader (San Jose, CA, USA) at a wavelength of 560 nm excitation/590 nm emission.

### 4.11. Statistical Analysis

All MIC measurements were performed in at least two independent experiments, each with three replicates. The MICs were calculated as an average of all measured values and represent the minimum concentrations which resulted in at least 80% inhibition of growth (MICPC_80_, MICBC_80_), metabolism (MICBM_80,_ MICMPB_80_) and biofilm formation (MICBF_80_), or resulted in to at least 80% reduction in preformed biofilms (MICBR_80_). The significance of the results was verified by *t*-test (*p* ≤ 0.05) using Statistica v13.5.0 (TIBCO Software Inc., Palo Alto, California).

The cytotoxicity results are expressed as the average IC_50_ ± standard error of the mean (SEM). Values of IC_50_ were obtained by using the online tool Quest Graph IC_50_ Calculator (AAT Bioquest Inc, Sunnyvale, CA, USA). One-way analysis of variance (ANOVA) was used, followed by Duncan’s post hoc test (*p* < 0.05) to show the differences between the groups. For ANOVA, the Statistica software (Tibco Software Inc., Palo Alto, CA, USA) was used in v12.

## 5. Conclusions

The aims of this study were to investigate the effectiveness of PdNPs and PtNPs against important food-borne pathogens and to evaluate their mechanisms of action. The interactions of NPs with bacteria were not dependent on their Gram-negative or Gram-positive characteristics. NPs bound to the bacterial cell wall and subsequently entered the cell through the wall and membrane, which resulted in bacterial disruption and leakage of intracellular components. In vitro cytotoxicity study confirmed that PdNPs and PtNPs did not exhibit any acute cytotoxicity. Both types of NPs were able to inhibit viable bacterial cells. However, the most significant antimicrobial effects were observed at the highest concentrations tested and seemed to be less effective for biofilm formation and its reduction. Hence, the regular use of NPs in food processing plants as an antimicrobial strategy may be challenging and potentially costly at this stage. Therefore, more studies are needed to elucidate the effects of NP size on antimicrobial efficacy and their potential chronic cytotoxicity prior to their application in the food industry.

## Figures and Tables

**Table 1:** Minimal concentrations inhibiting planktonic growth, biofilm formation and biofilm metabolic activity with their respective inhibitory rates of PdNPs for planktonic cells. Data represent the mean of at least three independent replicates. The significance of the results was verified by *t*-test (*p* ≤ 0.05).

BACTERIAL STRAIN	PdNPs (mg/L)					
	MICPC_80_	A_620_ Inhibition (%)	MICBF_80_	A_595_ Inhibition (%)	MICBM_80_	A_595_ Inhibition (%)
*S. aureus* 816	>22.25	61.4 ± 3.8	**22.25**	90.2 ± 4.1	>22.25	47.0 ± 16.6
*S. aureus* 1241	>22.25	61.4 ± 4.2	**22.25**	80.5 ± 2.0	>22.25	34.8 ± 16.2
*L. monocytogenes* 149	>22.25	51.0 ± 3.3	>22.25	14.8 ± 6.4	>22.25	18.1 ± 10.1
*L. monocytogenes* 164	>22.25	28.6 ± 6.9	>22.25	39.4 ± 4.6	>22.25	43.0 ± 11.5
*E. coli* 683/17	**22.25**	83.0 ± 0.6	>22.25	22.3 ± 1.2	>22.25	19.1 ± 0.6
*E. coli* 693/17	**22.25**	92.0 ± 0.1	>22.25	9.6 ± 4.1	>22.25	3.3 ± 0.3
*E. coli* 815	>22.25	66.0 ± 7.3	>22.25	47.0 ± 3.0	>22.25	35.8 ± 1.8
*E. coli* 859	>22.25	74.2 ± 0.1	>22.25	50.4 ± 2.4	>22.25	52.1 ± 2.8
*S.* Infantis S13	>22.25	41.1 ± 2.2	>22.25	17.0 ± 2.0	>22.25	11.0 ± 0.0
*S.* Infantis S59	>22.25	47.3 ± 0.8	>22.25	12.0 ± 1.8	>22.25	51.5 ± 2.3

PdNPs: 22.25, 11.13, 5.6, 2.8, 1.4, 0.7, 0.35, 0.18, 0.09, 0.05 mg/L (10 different concentrations); bold font marks the efficiency of ≥80 %; MICPC_80_ marks minimum inhibitory concentrations for planktonic growth; MICBF_80_ marks minimum inhibitory concentrations for biofilm formation; MICBM_80_ marks minimum inhibitory concentrations for biofilm metabolic activity.

**Table 2:** Minimal concentrations inhibiting planktonic growth, biofilm formation and biofilm metabolic activity with their respective inhibitory rates of PtNPs for planktonic cells. Data represent the mean of at least three independent replicates. The significance of the results was verified by *t*-test (*p* ≤ 0.05).

BACTERIAL STRAIN	PtNPs (mg/L)					
	MICPC_80_	A_620_ Inhibition (%)	MICBF_80_	A_595_ Inhibition (%)	MICBM_80_	A_595_ Inhibition (%)
*S. aureus* 816	>50.5	61.8 ± 5.6	**50.5**	88.8 ± 3.9	>50.5	53.7 ± 14.8
*S. aureus* 1241	>50.5	63.1 ± 5.9	>50.5	54.9 ± 7.5	>50.5	37.1 ± 6.1
*L. monocytogenes* 149	>50.5	49.2 ± 1.7	>50.5	12.7 ± 0.7	>50.5	21.5 ± 13.6
*L. monocytogenes* 164	>50.5	28.9 ± 5.7	>50.5	15.3 ± 0.3	>50.5	59.4 ± 10.6
*E. coli* 683/17	>50.5	76.8 ± 2.9	>50.5	21.2 ± 1.5	>50.5	5.8 ± 0.3
*E. coli* 693/17	>50.5	77.8 ± 1.6	>50.5	6.0 ± 1.7	>50.5	24.6 ± 8.9
*E. coli* 815	>50.5	67.6 ± 3.0	>50.5	5.3 ± 0.3	>50.5	32.3 ± 6.2
*E. coli* 859	>50.5	69.0 ± 7.2	>50.5	43.0 ± 5.4	>50.5	36.0 ± 4.9
*S.* Infantis S13	>50.5	51.6 ± 1.6	>50.5	19.4 ± 7.4	>50.5	23.8 ± 14.2
*S.* Infantis S59	>50.5	51.5 ± 1.9	>50.5	8.0 ± 0.2	>50.5	64.3 ± 0.7

PtNPs: 50.5, 25.25, 12.63, 6.3, 3.16, 1.58, 0.79, 0.39, 0.2, 0.1 mg/L (10 different concentrations); bold font marks the efficiency of ≥80 %; MICPC_80_ marks minimum inhibitory concentrations for planktonic growth; MICBF_80_ marks minimum inhibitory concentrations for biofilm formation; MICBM_80_ marks minimum inhibitory concentrations for biofilm metabolic activity.

**Table 3:** Minimal concentrations inhibiting further growth of biofilm cells, biofilm reduction and biofilm metabolic activity with their respective inhibitory rates of PdNPs for biofilms. Data represent the mean of at least three independent replicates. The significance of the results was verified by *t*-test (*p* ≤ 0.05).

BACTERIAL STRAIN	PdNPs (mg/L)					
	MICBC_80_	A_620_ Inhibition (%)	MICBR_80_	A_595_ Inhibition (%)	MICMPB_80_	A_595_ Inhibition (%)
*S. aureus* 816	**44.5**	98.0 ± 3.6	**44.5**	87.1 ± 7.4	**44.5**	96.5 ± 1.4
*S. aureus* 1241	**44.5**	94.0 ± 3.2	>44.5	75.7 ± 8.2	**44.5**	97.2 ± 0.6
*L. monocytogenes* 149	**44.5**	89.7 ± 5.2	>44.5	38.3 ± 0.3	**44.5**	100.0 ± 4.0
*L. monocytogenes* 164	**44.5**	85.3 ± 7.7	>44.5	29.1 ± 25.4	**44.5**	100.0 ± 0.6
*E. coli* 683/17	**22.25**	84.5 ± 5.6	>44.5	23.8 ± 11.7	**44.5**	100.0 ± 0.1
*E. coli* 693/17	**22.25**	86.6 ± 3.0	>44.5	20.3 ± 9.2	**44.5**	100.0 ± 0.2
*E. coli* 815	**22.25**	84.7 ± 3.8	>44.5	20.2 ± 3.8	**44.5**	93.3 ± 6.7
*E. coli* 859	**22.25**	90.5 ± 3.7	>44.5	52.5 ± 8.8	**44.5**	99.5 ± 1.0
*S.* Infantis S13	**44.5**	96.6 ± 0.1	**44.5**	83.6 ± 1.8	**44.5**	97.2 ± 0.0
*S.* Infantis S59	**44.5**	95.1 ± 0.2	**44.5**	82.7 ± 6.7	**44.5**	90.5 ± 2.1

PdNPs: 44.5, 22.25, 11.13, 5.6, 2.8, 1.4 mg/L (6 different concentrations); bold font marks the efficiency of ≥80%; MICBC_80_ marks minimum inhibitory concentrations for further growth of biofilm cells; MICBR_80_ marks minimum inhibitory concentrations for biofilm reduction; MICMPB_80_ marks minimum inhibitory concentrations for metabolic activity of preformed biofilm.

**Table 4:** Minimal concentrations inhibiting further growth of biofilm cells, biofilm reduction and biofilm metabolic activity with their respective inhibitory rates of PtNPs for biofilms. Data represent the mean of at least three independent replicates. The significance of the results was verified by *t*-test (*p* ≤ 0.05).

BACTERIAL STRAIN	PtNPs (mg/L)					
	MICBC_80_	A_620_ Inhibition (%)	MICBR_80_	A_595_ Inhibition (%)	MICMPB_80_	A_595_ Inhibition (%)
*S. aureus* 816	**101**	87.3 ± 5.4	**101**	84.5 ± 0.5	**101**	100.0 ± 0.0
*S. aureus* 1241	**101**	87.2 ± 4.6	**101**	83.0 ± 2.3	**101**	100.0 ± 0.0
*L. monocytogenes* 149	**101**	98.8 ± 2.3	>101	34.2 ± 5.2	**101**	99.6 ± 15.6
*L. monocytogenes* 164	**101**	95.8 ± 1.9	>101	68.1 ± 12.9	**101**	97.2 ± 2.8
*E. coli* 683/17	**50.5**	78.0 ± 4.5	>101	14.2 ± 24.3	**101**	98.9 ± 2.2
*E. coli* 693/17	**50.5**	80.7 ± 2.3	>101	15.6 ± 21.2	**101**	97.8 ± 2.2
*E. coli* 815	**101**	95.4 ± 0.2	>101	3.9 ± 6.6	**101**	87.6 ± 12.4
*E. coli* 859	**50.5**	81.8 ± 2.9	>101	53.9 ± 1.4	**101**	100.0 ± 0.0
*S.* Infantis S13	**101**	98.9 ± 0.1	>101	31.5 ± 27.0	**101**	100.0 ± 0.0
*S.* Infantis S59	**101**	99.1 ± 0.5	>101	42.2 ± 13.2	**101**	100.0 ± 2.5

PtNPs: 101, 50.5, 25.25, 12.63, 6.3, 3.16 mg/L (6 different concentrations); bold font marks the efficiency of ≥80%; MICBC_80_ marks minimum inhibitory concentrations for further growth of biofilm cells; MICBR_80_ marks minimum inhibitory concentrations for biofilm reduction; MICMPB_80_ marks minimum inhibitory concentrations for metabolic activity of preformed biofilm.

**Table 5:** Reduction in viable colony counts caused by 24 h exposure to PdNPs (22.25 mg/L). Data represent the mean of at least four independent replicates.

BACTERIAL STRAIN	Control Log (CFU/mL)	PdNPs (22.25 mg/L) Log (CFU/mL)	Log_10_ CFU Reduction	Inhibitory Rate (%)
*S. aureus* 816	9.8 ± 0.0	9.0 ± 0.2	0.8 ± 0.2	81.5 ± 1.3
*S. aureus* 1241	10.6 ± 0.1	8.2 ± 0.2	2.4 ± 0.2	99.0 ± 0.4
*L. monocytogenes* 149	9.1 ± 0.0	8.8 ± 0.0	0.3 ± 0.0	55.2 ± 0.2
*L. monocytogenes* 164	10.6 ± 0.0	9.6 ± 0.1	1.0 ± 0.1	89.3 ± 9.8
*E. coli* 683/17	9.6 ± 0.0	8.7 ± 0.0	0.9 ± 0.0	87.3 ± 0.1
*E. coli* 693/17	10.4 ± 0.1	8.8 ± 0.1	1.6 ± 0.1	97.0 ± 2.0
*E. coli* 815	10.6 ± 0.1	8.9 ± 0.1	1.7 ± 0.1	97.5 ± 1.5
*E. coli* 859	10.8 ± 0.0	9.8 ± 0.1	1.0 ± 0.1	91.2 ± 7.9
*S.* Infantis S13	10.9 ± 0.0	9.7 ± 0.0	1.2 ± 0.0	93.3 ± 0.1
*S.* Infantis S59	10.9 ± 0.0	9.9 ± 0.1	1.0 ± 0.1	89.3 ± 9.8

**Table 6:** Reduction in viable colony counts caused by 24 h exposure to PtNPs (50.5 mg/L). Data represent the mean of at least four independent replicates.

BACTERIAL STRAIN	Control Log (CFU/mL)	PtNPs (50.5 mg/L) Log (CFU/mL)	Log_10_ CFU Reduction	Inhibitory Rate (%)
*S. aureus* 816	9.8 ± 0.0	8.1 ± 0.0	1.7 ± 0.0	97.8 ± 0.2
*S. aureus* 1241	10.6 ± 0.1	8.6 ± 0.1	2.0 ± 0.1	99.0 ± 0.4
*L. monocytogenes* 149	9.1 ± 0.0	8.3 ± 0.1	0.8 ± 0.1	83.8 ± 1.6
*L. monocytogenes* 164	10.6 ± 0.0	9.7 ± 0.1	0.9 ± 0.1	89.3 ± 0.1
*E. coli* 683/17	9.6 ± 0.0	8.7 ± 0.1	0.9 ± 0.1	88.2 ± 0.3
*E. coli* 693/17	10.4 ± 0.1	8.8 ± 0.1	1.6 ± 0.1	97.5 ± 0.4
*E. coli* 815	10.6 ± 0.1	8.7 ± 0.1	1.9 ± 0.1	98.5 ± 0.4
*E. coli* 859	10.8 ± 0.0	9.0 ± 0.1	1.8 ± 0.1	98.4 ± 0.0
*S.* Infantis S13	10.9 ± 0.0	9.7 ± 0.0	1.2 ± 0.0	94.0 ± 0.0
*S.* Infantis S59	10.9 ± 0.0	9.9 ± 0.0	1.0 ± 0.0	90.7 ± 0.1

**Table 7:** Cytotoxicity of nanoparticles expressed as a concentration halving the viability of cell lines (IC_50_). The data are presented as an average of 3 repetitions with SEM.

	IC_50_ (mg/L)	
Cell Lines	PdNPs	PtNPs
HRTEC	>4.45	>10.1
HaCat	>4.45	>10.1
HDF	>4.45	>10.1
HEK 293	>4.45	>10.1
HCAEC	>4.45	>10.1

Cell lines: primary human renal tubular epithelial cells (HRTECs), human keratinocytes (HaCat), human dermal fibroblasts (HDFs), human epithelial kidney cells (HEK 293), primary human coronary artery endothelial cells (HCAECs).

**Table 8:** List of bacterial strains and their origin.

Bacterial Strain	Origin
*Staphylococcus aureus* 816	Sea fish
*Staphylococcus aureus* 1241	Cow milk
*Listeria monocytogenes* 149	Pork ham
*Listeria monocytogenes* 164	Pork ham
*Escherichia coli* 683/17	Salt bath (cheese industry)
*Escherichia coli* 693/17	Floor (cheese industry)
*Escherichia coli* 815	Cheese packaging
*Escherichia coli* 859	Cheese packaging
*Salmonella enterica* Infantis S13	Wastewater treatment
*Salmonella enterica* Infantis S59	Frozen chicken meat

## Data Availability

Data are available on request to the corresponding author.

## Supplementary Materials

The following are available online at https://www.mdpi.com/article/10.3390/ijms22157892/s1, Table S1: Absorbance values (A_620_) of the effect of PdNPs on planktonic growth., Figure S1: Inhibition effect of PdNPs on planktonic growth. Figure S2: Inhibition effect of PdNPs on biofilm formation. Figure S3: Quantification of biofilm formation with the use of 10 different PdNPs concentrations. Table S2: Absorbance values (A_620_) of the effect of PdNPs on further growth of biofilm cells. Figure S4: Inhibition effect of PdNPs on further growth of biofilm cells. Figure S5: Reduction effect of PdNPs on preformed biofilms. Figure S6: Quantification of biofilm reduction with the use of 6 different PdNPs concentrations. Table S3: Absorbance values (A_620_) of the effect of PtNPs on planktonic growth. Figure S7: Inhibition effect of PtNPs on planktonic growth. Figure S8: Inhibition effect of PtNPs on biofilm formation. Figure S9: Quantification of biofilm formation with the use of 10 different PtNPs concentrations. Table S4: Absorbance values (A_620_) of the effect of PtNPs on further growth of biofilm cells. Figure S10: Inhibition effect of PtNPs on further growth of biofilm cells. Figure S11: Reduction effect of PtNPs on preformed biofilms. Figure S12: Quantification of biofilm reduction with the use of 6 different PtNPs concentrations.
