# Supplementary material for: Antimicrobial Properties of Palladium and Platinum Nanoparticles: A New Tool for Combating Food-Borne Pathogens
Source: Int J Mol Sci. 2021 Jul 23;22(15):7892. doi: 10.3390/ijms22157892 (PMC8346086; doi:10.3390/ijms22157892)

Supplementary Materials

Antimicrobial Properties of Palladium and Platinum Nanoparticles: A New Tool for Combating Food-Borne Pathogens

Ondrej Chlumsky*[1], Sabina Purkrtova[1], Hana Michova[1], Hana Sykorova[1], Petr Slepicka[2], Dominik Fajstavr[2], Pavel Ulbrich[1], Jitka Viktorova[1] and Katerina Demnerova[1]

[1] Department of Biochemistry and Microbiology, University of Chemistry and Technology, Technicka 5, 166 28, Prague 6, Czech Republic
[2] Department of Solid State Engineering, University of Chemistry and Technology, Technicka 5, 166 28, Prague 6, Czech Republic
* Correspondence: chlumsko@vscht.cz

Table S1. Absorbance values (A_{620}) of the effect of PdNPs on planktonic growth. Data represent the mean of independent experiments (n = 2), each with three technical replicates.

PdNPs	22.25 mg/L A_{620}	11.13 mg/L A_{620}	5.6 mg/L A_{620}	2.8 mg/L A_{620}	1.4 mg/L A_{620}	0.7 mg/L A_{620}	0.35 mg/L A_{620}	0.18 mg/L A_{620}	0.09 mg/L A_{620}	0.05 mg/L A_{620}	CONTROL
SA 816	0.2408	0.4382	0.5253	0.5560	0.5743	0.5723	0.5795	0.6152	0.5873	0.5837	0.5938
SA 1241	0.1798	0.3377	0.4312	0.4613	0.4850	0.4755	0.4878	0.4882	0.4635	0.4808	0.4922
LM 149	0.1265	0.1932	0.2265	0.2520	0.2485	0.2817	0.2692	0.2628	0.2777	0.2557	0.2682
LM 164	0.1902	0.2598	0.2682	0.2708	0.2708	0.2815	0.2860	0.2817	0.2687	0.2803	0.2810
EC 683/17	0.1005	0.2543	0.3667	0.4453	0.4987	0.5500	0.5660	0.5895	0.6000	0.6095	0.5807
EC 693/17	0.0153	0.0923	0.1310	0.1153	0.1540	0.1547	0.1510	0.1557	0.1700	0.1737	0.1823
EC 815	0.1977	0.4103	0.4965	0.6088	0.6295	0.7277	0.7350	0.6847	0.7367	0.6832	0.6575
EC 859	0.1433	0.2830	0.4078	0.4652	0.5030	0.5278	0.5607	0.5973	0.5870	0.5780	0.5563
SAL 13	0.8120	1.1387	1.2795	1.3348	1.3647	1.3860	1.3917	1.3992	1.3898	1.3962	1.4045
SAL 59	0.5925	0.8457	0.9958	1.0710	1.0898	1.1157	1.1255	1.1232	1.1315	1.1275	1.1322

Figure S1. Inhibition effect of PdNPs on planktonic growth. Data represent the mean of independent experiments (n = 2), each with three technical replicates.

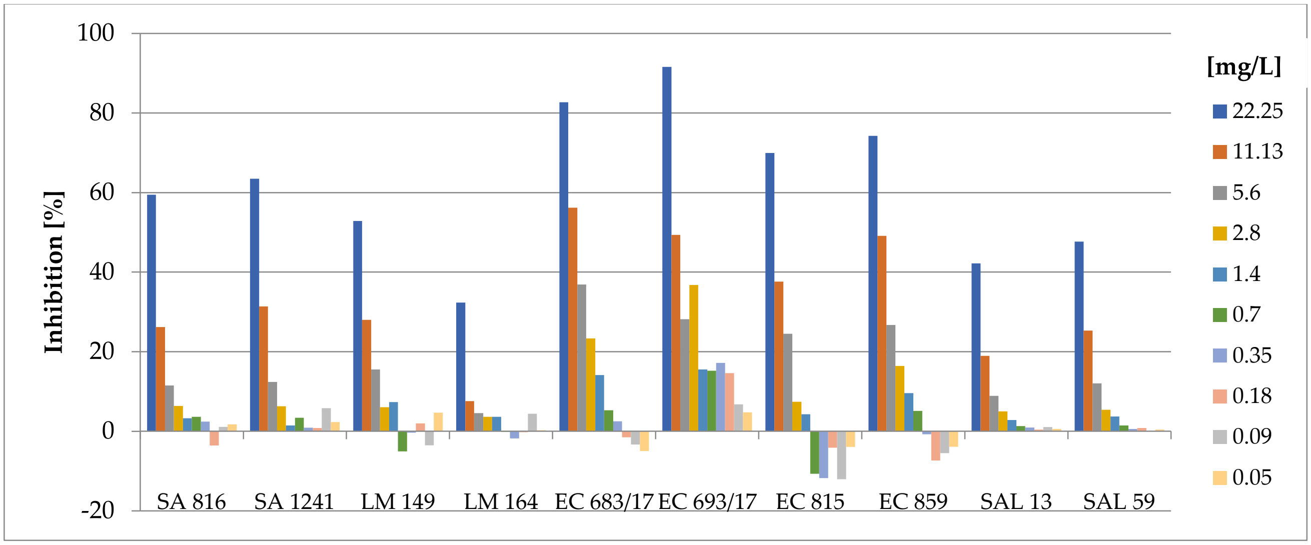

Figure S2. Inhibition effect of PdNPs on biofilm formation. Data represent the mean of independent experiments (n = 2), each with three technical replicates.

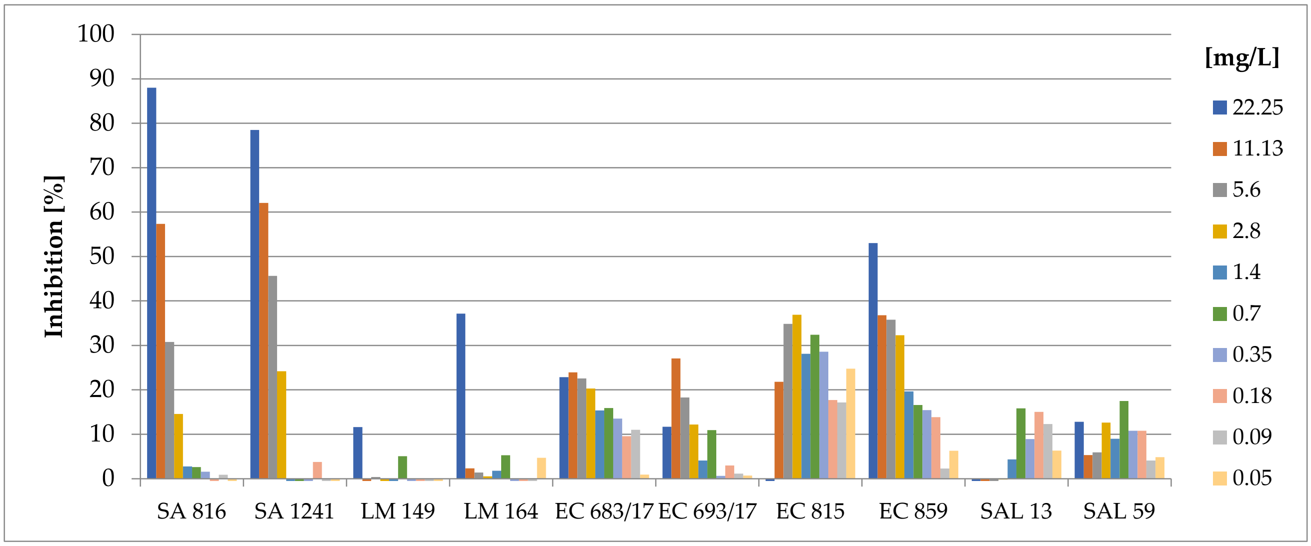

Figure S3. Quantification of biofilm formation with the use of 10 different PdNPs concentrations. Red frame marks minimal inhibitory concentrations able to prevent biofilm formation by at least 80 %.

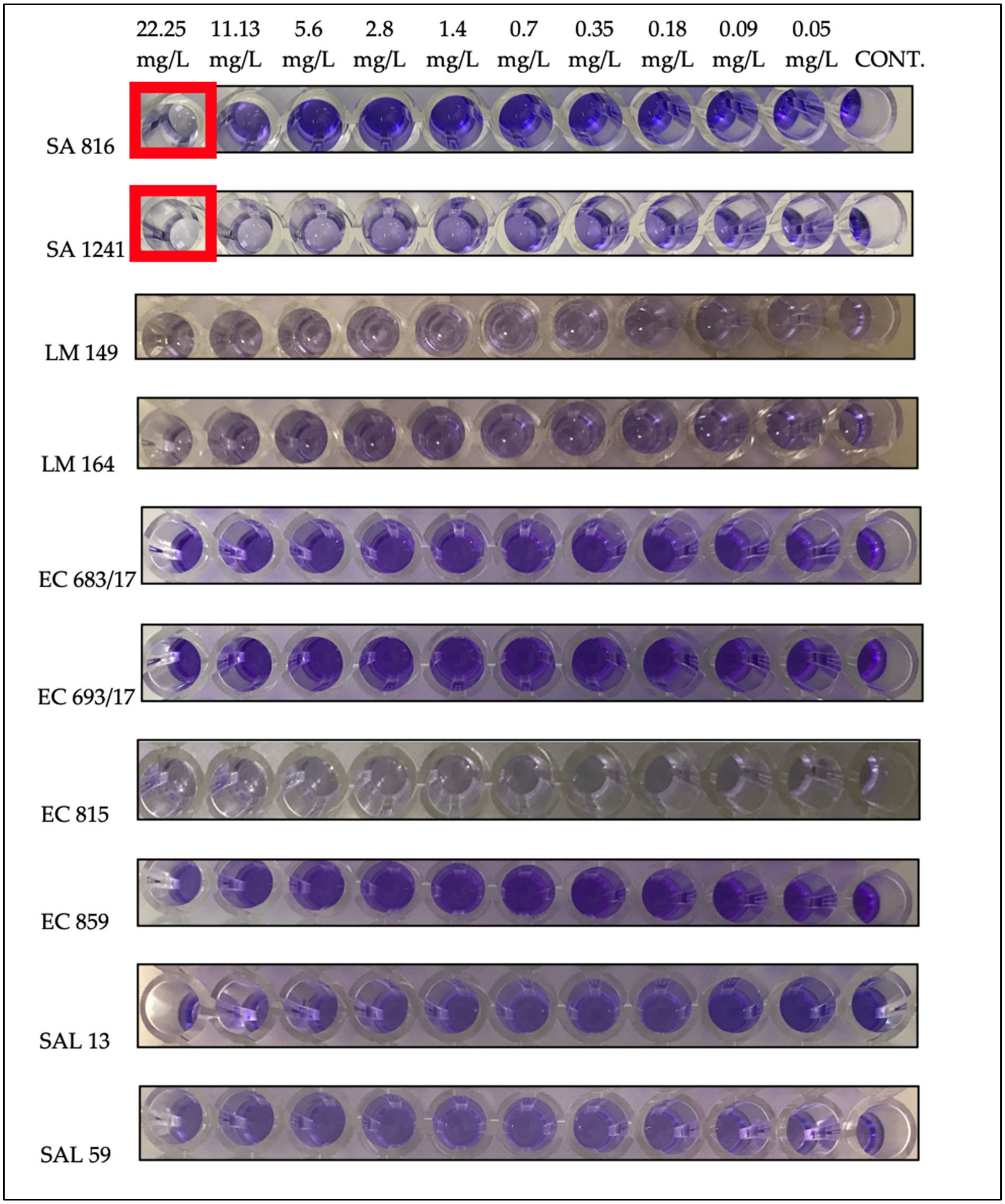

Table S2. Absorbance values (A_{620}) of the effect of PdNPs on further growth of biofilm cells. Data represent the mean of independent experiments (n = 2), each with three technical replicates.

PdNPs	44.5 mg/L A_{620}	22.25 mg/L A_{620}	11.13 mg/L A_{620}	5.6 mg/L A_{620}	2.8 mg/L A_{620}	1.4 mg/L A_{620}	CONTROL
SA 816	0.0117	0.1070	0.2078	0.3467	0.2697	0.3318	0.3303
SA 1241	0.0150	0.1375	0.2520	0.3360	0.3208	0.3620	0.3372
LM 149	0.0150	0.0750	0.1093	0.1303	0.1237	0.1253	0.1923
LM 164	0.0130	0.0545	0.1173	0.1022	0.1322	0.1093	0.1242
EC 683/17	0.0233	0.0488	0.1372	0.1903	0.2238	0.2610	0.3850
EC 693/17	0.0535	0.0618	0.1542	0.2015	0.2492	0.2753	0.4143
EC 815	0.0655	0.1115	0.3273	0.4693	0.5113	0.5557	0.6513
EC 859	0.1082	0.0215	0.1177	0.1895	0.2068	0.2420	0.2828
SAL 13	0.0432	0.7045	1.0032	1.1412	1.2037	1.2295	1.2903
SAL 59	0.0513	0.6085	0.8298	0.9402	1.0228	0.9882	1.0303

Figure S4. Inhibition effect of PdNPs on further growth of biofilm cells. Data represent the mean of independent experiments (n = 2), each with three technical replicates.

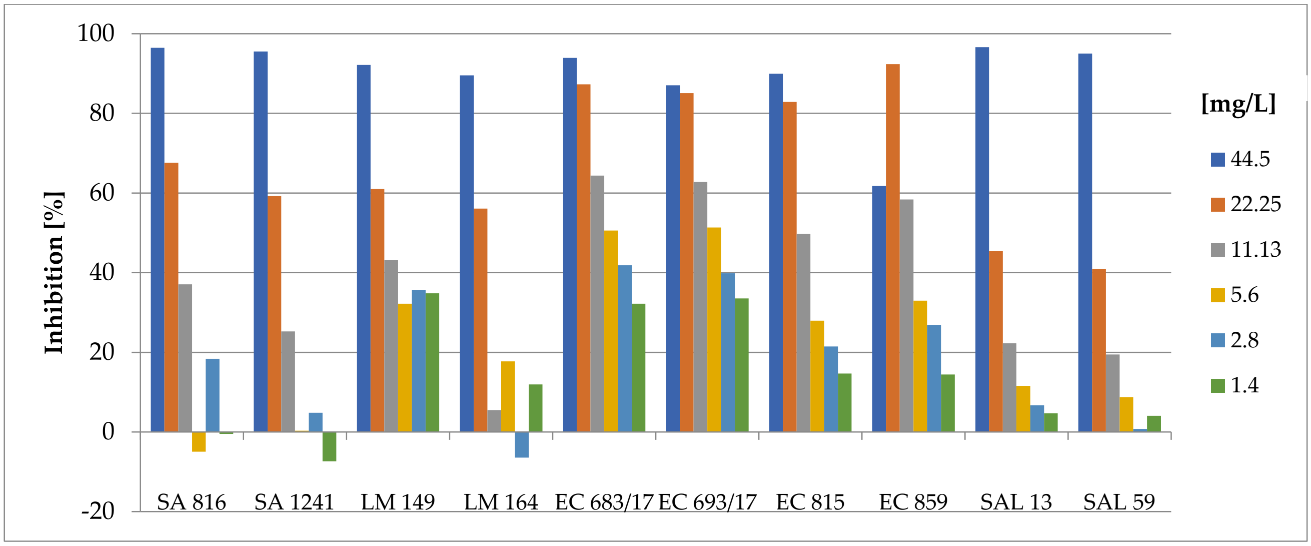

Figure S5. Reduction effect of PdNPs on preformed biofilms. Data represent the mean of independent experiments (n = 2), each with three technical replicates.

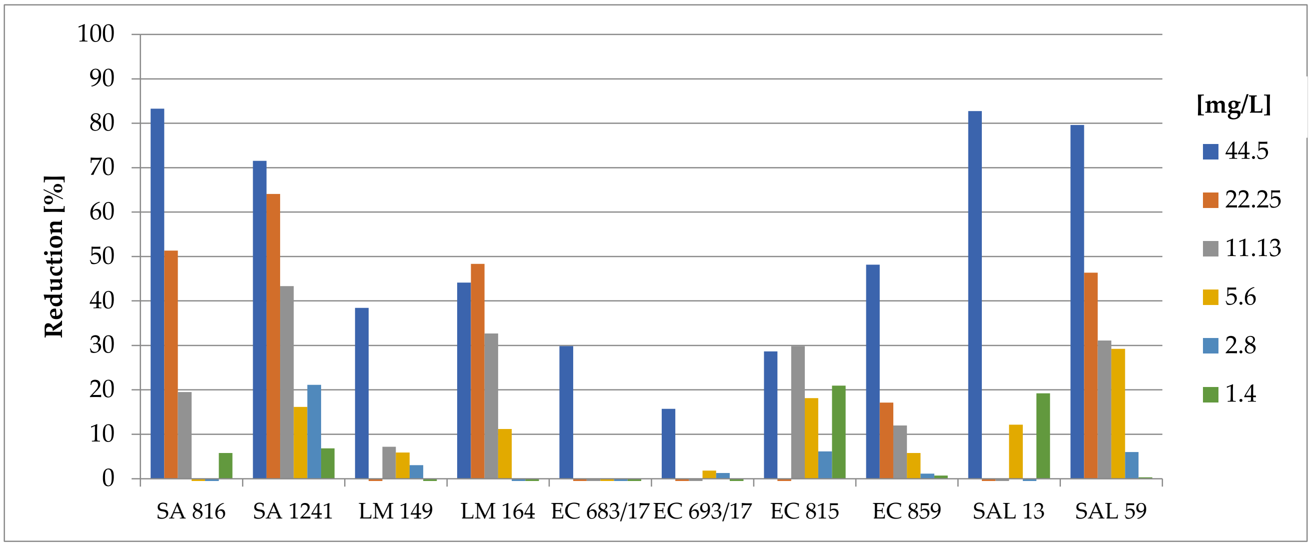

Figure S6. Quantification of biofilm reduction with the use of 6 different PdNPs concentrations. Red frame marks minimal inhibitory concentrations able to reduce a preformed biofilm by at least 80 %.

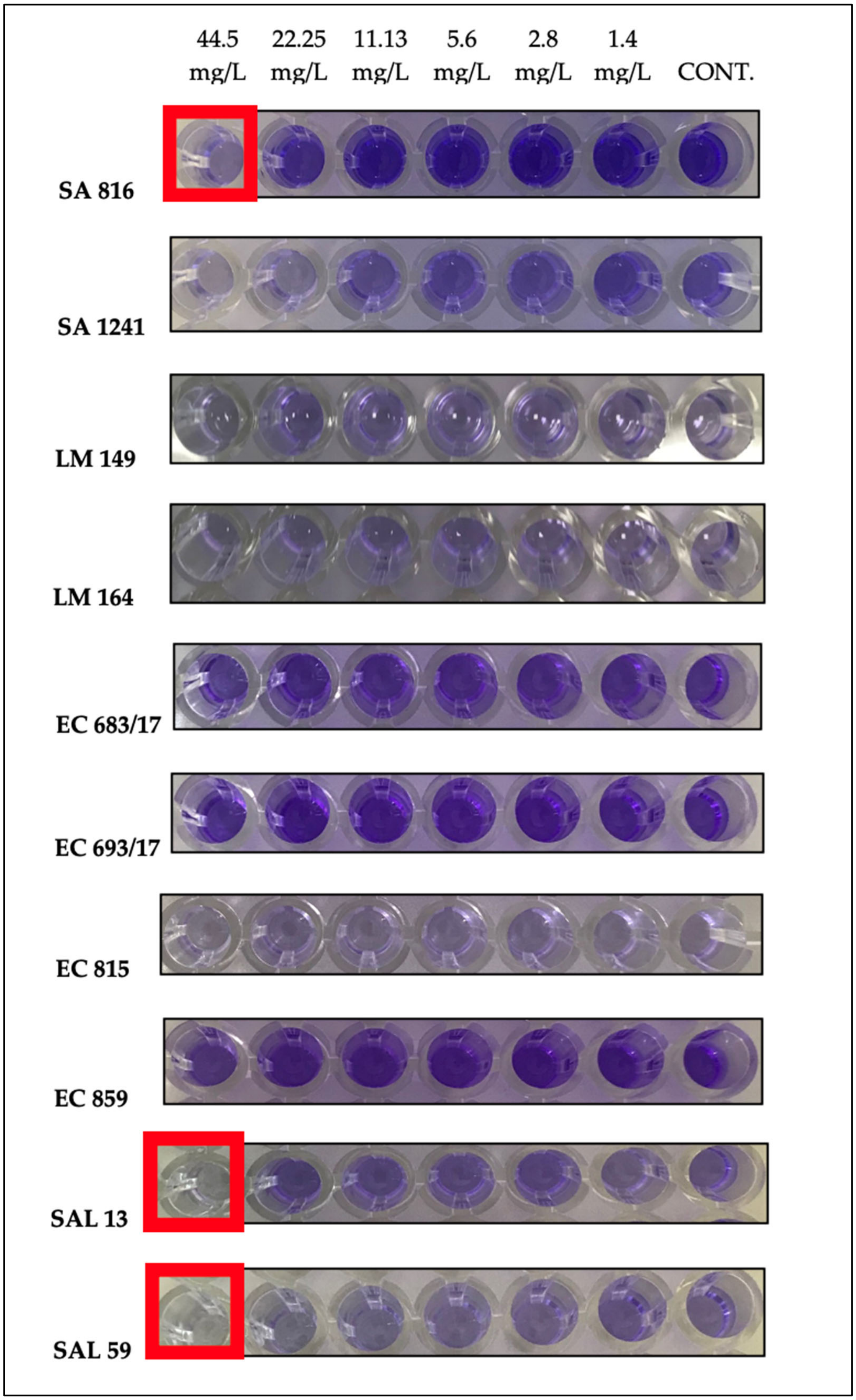

Table S3. Absorbance values (A_{620}) of the effect of PtNPs on planktonic growth. Data represent the mean of independent experiments (n = 2), each with three technical replicates.

PtNPs	50.5 mg/L A_{620}	25.25 mg/L A_{620}	12.63 mg/L A_{620}	6.3 mg/L A_{620}	3.16 mg/L A_{620}	1.58 mg/L A_{620}	0.79 mg/L A_{620}	0.39 mg/L A_{620}	0.2 mg/L A_{620}	0.1 mg/L A_{620}	CONTROL
SA 816	0.2450	0.4528	0.5290	0.5690	0.5773	0.5902	0.6005	0.5995	0.6025	0.6113	0.5967
SA 1241	0.1592	0.3180	0.4120	0.4582	0.4475	0.4695	0.4702	0.4910	0.4905	0.4925	0.4003
LM 149	0.1248	0.1925	0.2258	0.2405	0.2552	0.2655	0.2590	0.2692	0.2645	0.2692	0.2415
LM 164	0.1757	0.2288	0.2505	0.2592	0.2528	0.2688	0.2497	0.2532	0.2455	0.2477	0.2433
EC 683/17	0.1260	0.2485	0.3513	0.4330	0.4857	0.5250	0.5690	0.6112	0.6012	0.6083	0.5802
EC 693/17	0.1078	0.2143	0.3057	0.3800	0.4187	0.4505	0.4933	0.5212	0.5357	0.5433	0.5057
EC 815	0.2328	0.4895	0.5882	0.7142	0.7003	0.7805	0.7860	0.7668	0.8045	0.7993	0.7520
EC 859	0.1462	0.2987	0.4128	0.5042	0.5373	0.5597	0.5675	0.5752	0.5753	0.5773	0.5375
SAL 13	0.6757	0.9488	1.1242	1.2778	1.3187	1.3595	1.3710	1.3745	1.3783	1.3765	1.3735
SAL 59	0.5668	0.7992	0.9937	1.1165	1.1580	1.1835	1.1965	1.1970	1.1988	1.1935	1.1913

Figure S7. Inhibition effect of PtNPs on planktonic growth. Data represent the mean of independent experiments (n = 2), each with three technical replicates.

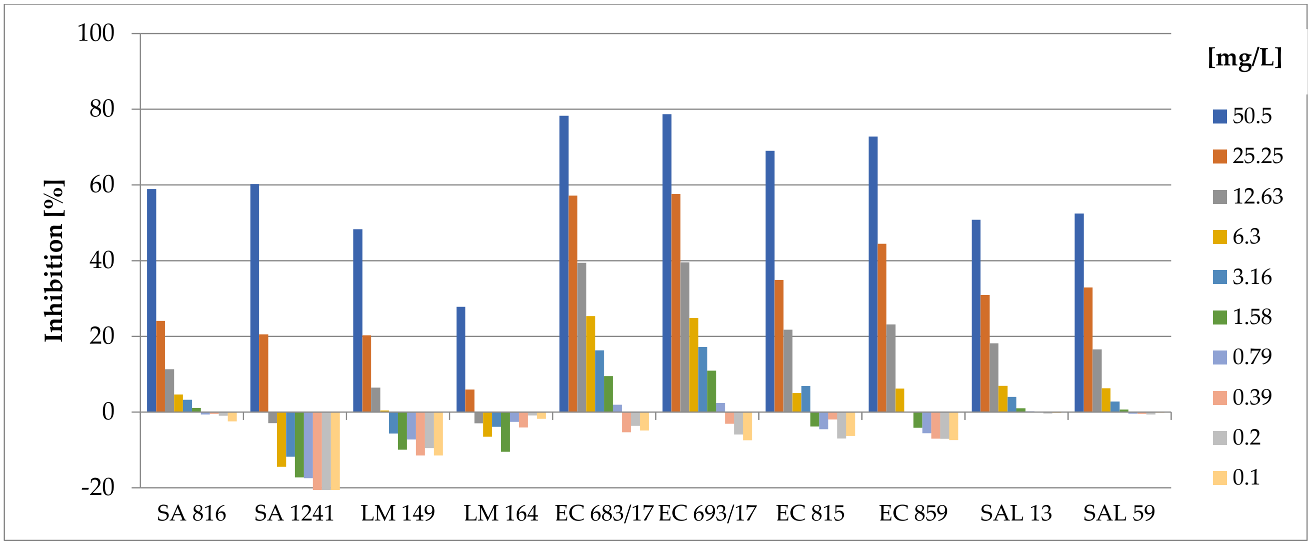

Figure S8. Inhibition effect of PtNPs on biofilm formation. Data represent the mean of independent experiments (n = 2), each with three technical replicates.

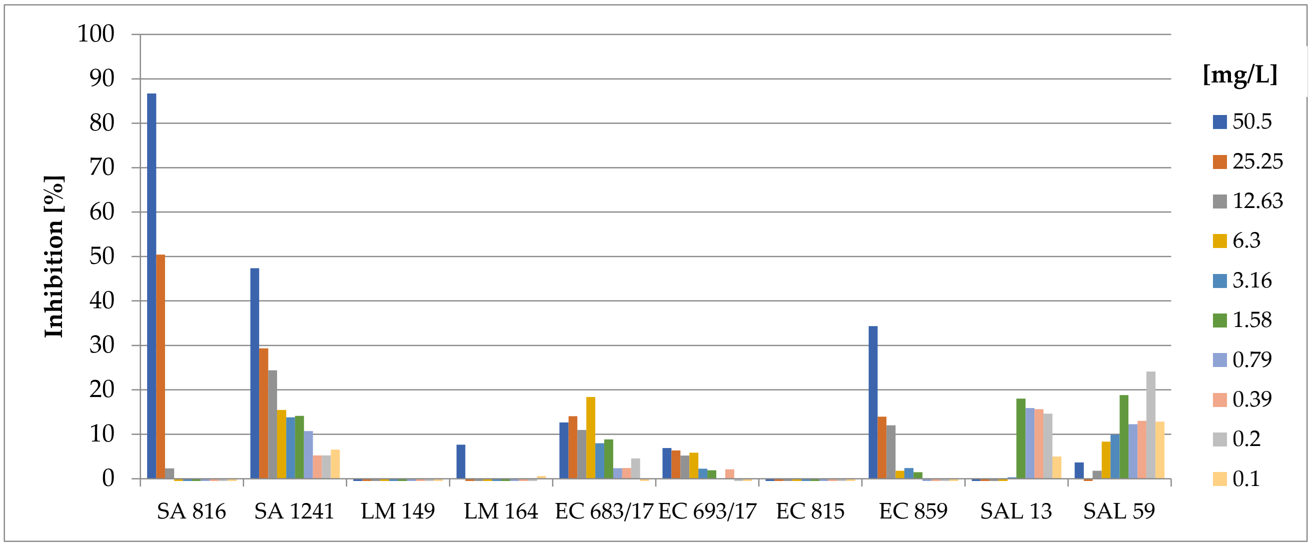

Figure S9. Quantification of biofilm formation with the use of 10 different PtNPs concentrations. Red frame marks minimal inhibitory concentrations able to prevent biofilm formation by at least 80 %.

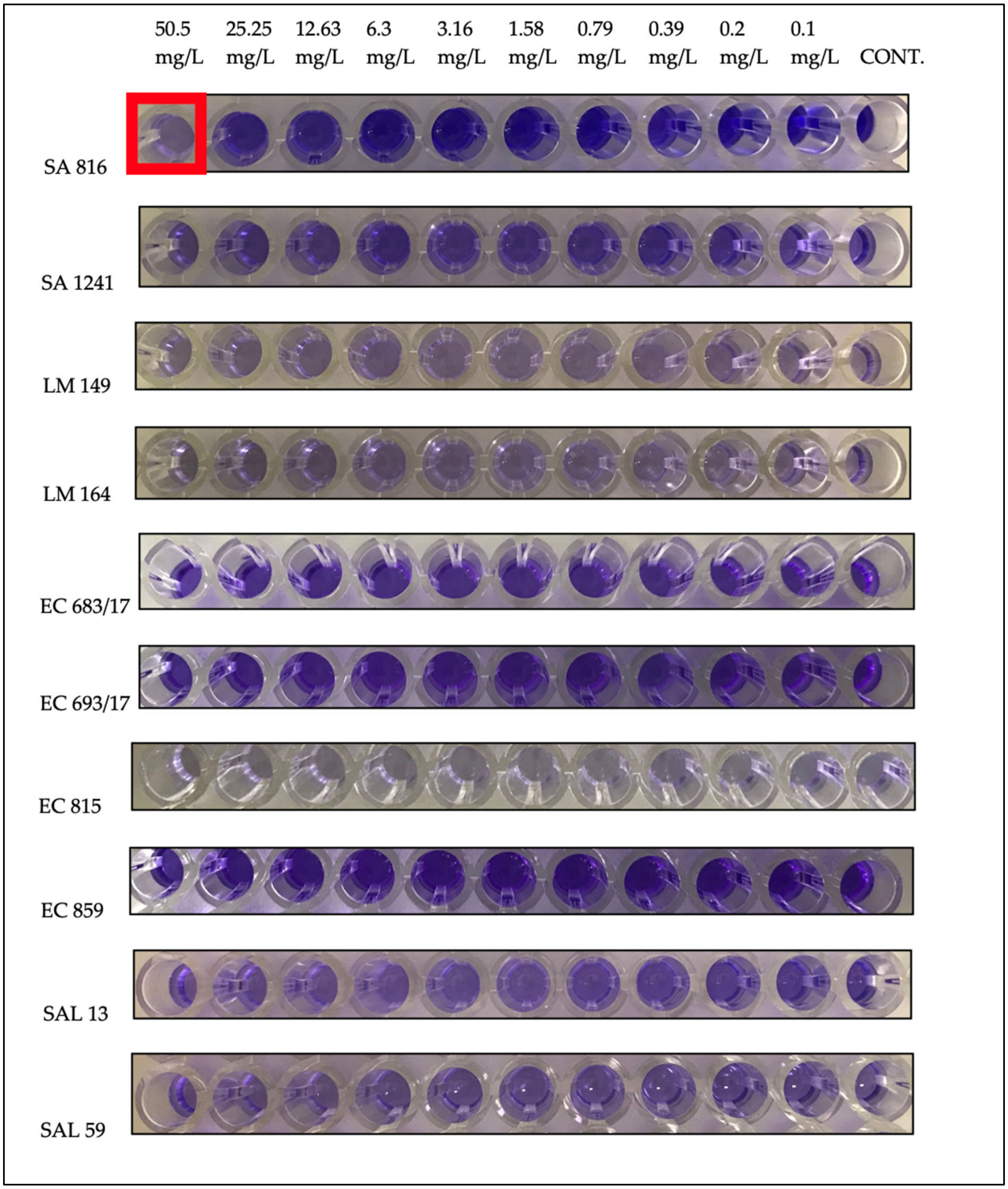

Table S4. Absorbance values (A_{620}) of the effect of PtNPs on further growth of biofilm cells. Data represent the mean of independent experiments (n = 2), each with three technical replicates.

PtNPs	101 mg/L A_{620}	50.5 mg/L A_{620}	25.25 mg/L A_{620}	12.63 mg/L A_{620}	6.3 mg/L A_{620}	3.16 mg/L A_{620}	CONTROL
SA 816	0.0488	0.1282	0.1897	0.2372	0.3027	0.2873	0.3225
SA 1241	0.0510	0.1483	0.2075	0.2720	0.3167	0.3072	0.3367
LM 149	0.0025	0.0517	0.0665	0.0803	0.0773	0.0792	0.1085
LM 164	0.0075	0.0828	0.1448	0.1305	0.1257	0.1077	0.1473
EC 683/17	0.0263	0.0798	0.1508	0.2068	0.2552	0.2778	0.4020
EC 693/17	0.0260	0.0797	0.1485	0.1962	0.2463	0.2690	0.3900
EC 815	0.0292	0.2185	0.3707	0.4668	0.5557	0.5728	0.6502
EC 859	0.0405	0.0603	0.1265	0.1678	0.2133	0.2333	0.3082
SAL 13	0.0137	0.5822	0.8712	1.0373	1.0858	1.1347	1.1795
SAL 59	0.0068	0.4560	0.7332	0.8052	0.9032	0.9120	0.9823

Figure S10. Inhibition effect of PtNPs on further growth of biofilm cells. Data represent the mean of independent experiments (n = 2), each with three technical replicates.

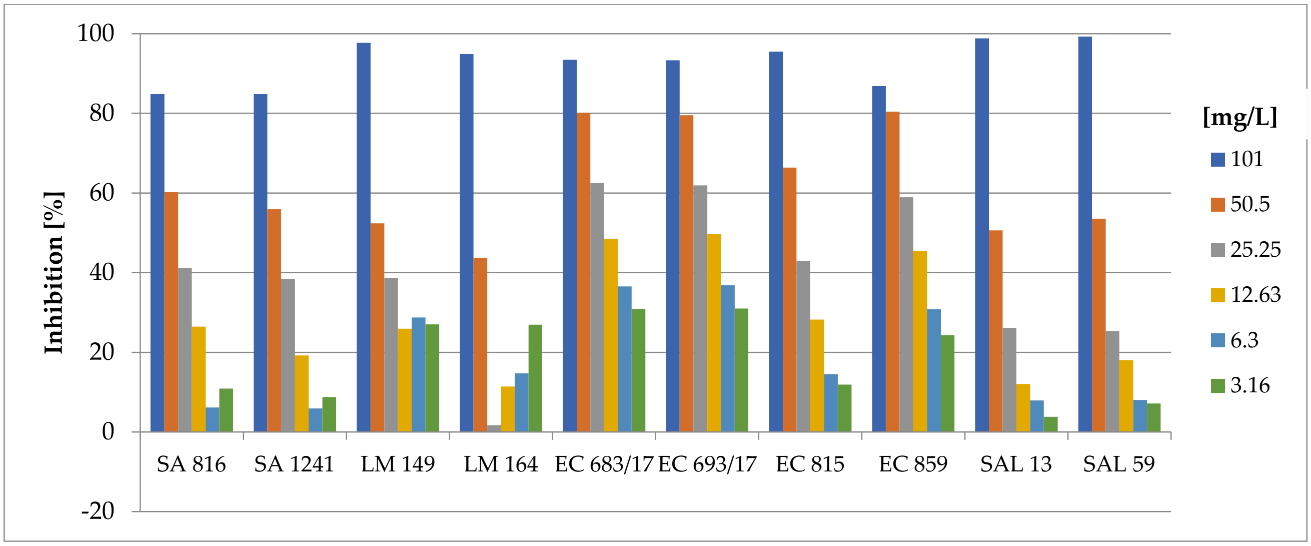

Figure S11. Reduction effect of PtNPs on preformed biofilms. Data represent the mean of independent experiments (n = 2), each with three technical replicates.

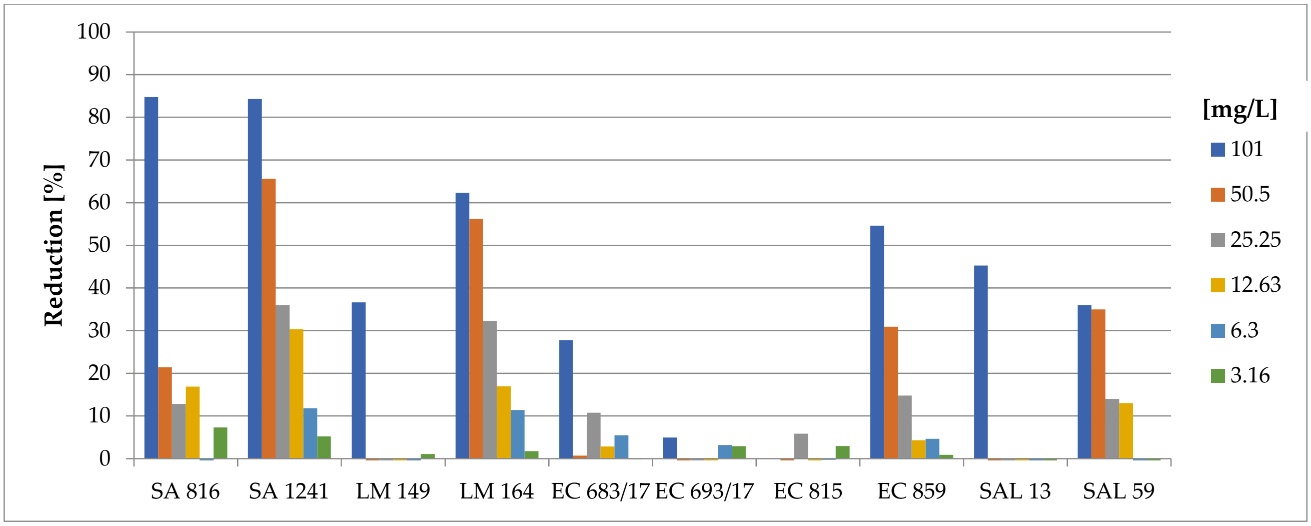

Figure S12. Quantification of biofilm reduction with the use of 6 different PtNPs concentrations. Red frame marks minimal inhibitory concentrations reduce a preformed biofilm by at least 80 %.

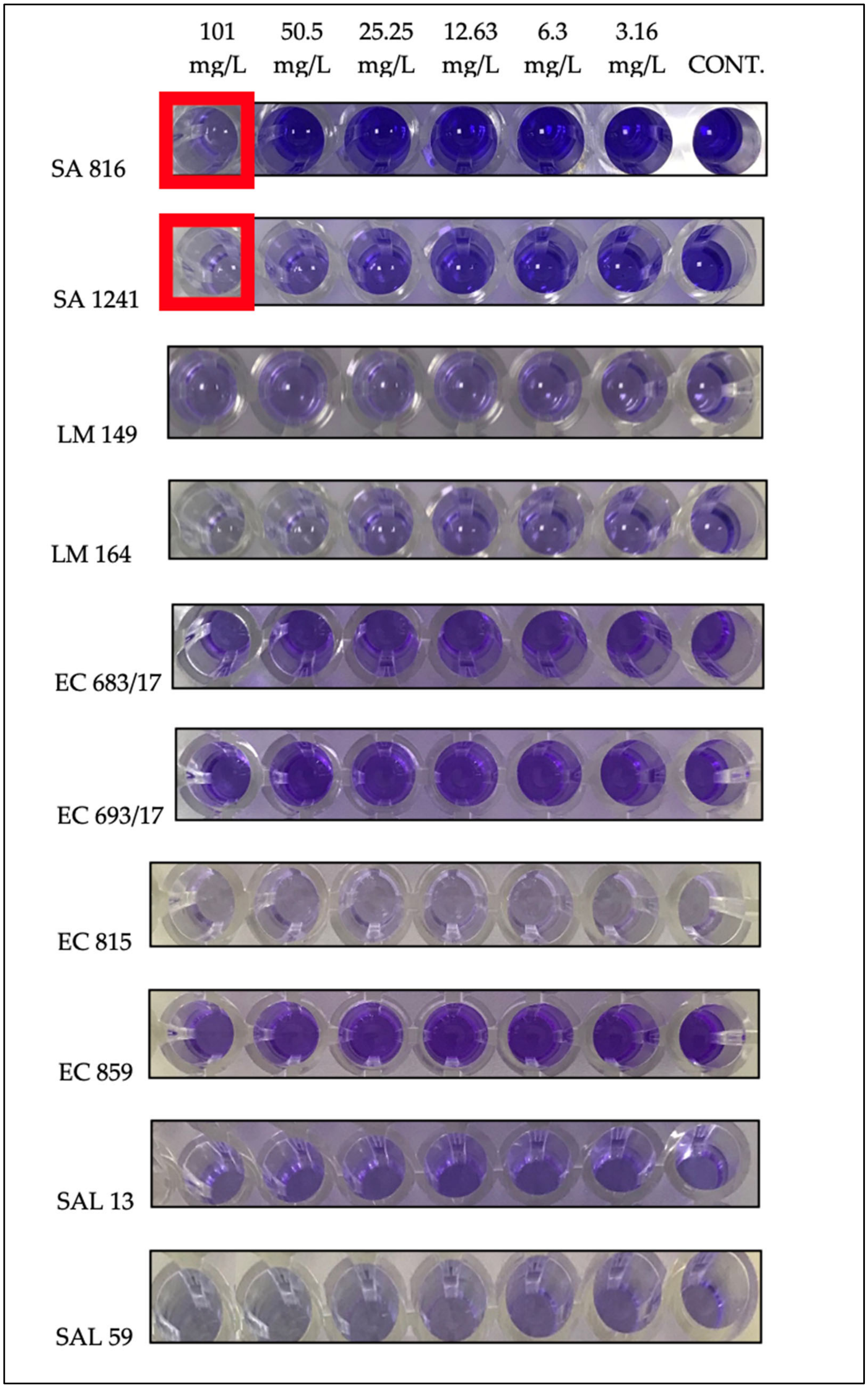

Supplement: Supplementary file 1 [file ijms-22-07892-s001.zip › ijms-1296657-supplementary.pdf]
